# Fatty acid composition and biophysical characteristics of the cell membrane of feline spermatozoa

**DOI:** 10.1038/s41598-024-61006-5

**Published:** 2024-05-03

**Authors:** Sylwia Prochowska, Dorota Bonarska-Kujawa, Łukasz Bobak, Maria Eberhardt, Wojciech Niżański

**Affiliations:** 1 Department of Reproduction and Clinic of Farm Animals, Wrocław University of Environmental and Life Sciences, 50-366 Wrocław, Poland; 2 Department of Physics and Biophysics, Wrocław University of Environmental and Life Sciences, 50-375 Wrocław, Poland; 3 Department of Functional Food Product Development, Wrocław University of Environmental and Life Sciences, 51-630 Wrocław, Poland

**Keywords:** Domestic cat, Semen, Fatty acids, Membrane fluidity, Membrane generalized potential, Biochemistry, Lipids, Fatty acids, Biophysics, Membrane biophysics

## Abstract

Sperm membrane composition and biophysical characteristics play a pivotal role in many physiological processes (i.e. sperm motility, capacitation, acrosome reaction and fusion with the oocyte) as well as in semen processing (e.g. cryopreservation). The aim of this study was to characterize the fatty acid content and biophysical characteristics (anisotropy, generalized polarization) of the cell membrane of domestic cat spermatozoa. Semen was collected from 34 adult male cats by urethral catheterization. After a basic semen evaluation, the fatty acid content of some of the samples (n = 11) was evaluated by gas chromatography. Samples from other individuals (n = 23) were subjected to biophysical analysis: membrane anisotropy (which is inversely proportional to membrane fluidity) and generalized polarization (describing lipid order); both measured by fluorimetry at three temperature points: 38 °C, 25 °C and 5 °C. Spermatozoa from some samples (n = 10) were cryopreserved in TRIS egg yolk-glycerol extender and underwent the same biophysical analysis after thawing. Most fatty acids in feline spermatozoa were saturated (69.76 ± 24.45%), whereas the polyunsaturated fatty acid (PUFA) content was relatively low (6.12 ± 5.80%). Lowering the temperature caused a significant decrease in membrane fluidity and an increase in generalized polarization in fresh spermatozoa, and these effects were even more pronounced following cryopreservation. Anisotropy at 38 °C in fresh samples showed strong positive correlations with viability and motility parameters after thawing. In summary, feline spermatozoa are characterized by a very low PUFA content and a low ratio of unsaturated:saturated fatty acids, which may contribute to low oxidative stress. Cryopreservation alters the structure of the sperm membrane, increasing the fluidity of the hydrophobic portion of the bilayer and the lipid order in the hydrophilic portion. Because lower membrane fluidity in fresh semen was linked with better viability and motility after cryopreservation, this parameter may be considered an important factor in determination of sperm cryoresistance.

## Introduction

Male fertility depends on the proper functioning of the spermatozoa, in which the structural integrity and dynamic properties of the sperm membranes play a pivotal role. The sperm plasma membrane not only serves as a protective barrier, but also actively contributes to various physiological processes essential for successful fertilization, i.e., sperm motility, capacitation, acrosome reaction, and, finally, fusion with the oocyte^1,2^. The studies on sperm membrane biochemistry began in the second half of the XIX century and identified the main lipid classes: phospholipids, glycolipids, and sterols^3^. Subsequent studies provided a deep insight into the composition of the membrane, and the precise lipid molecular content is known for many species, including human^4^, farm animals ^5–10^, horses^11^, poultry^5^ and even exotic animals such as elephants^12^, bats^13^, and marsupials^14^. However, for cats, there has been only one publication and one conference abstract, in which qualitative analysis was performed by matrix-assisted laser desorption and ionization time-of-flight mass spectrometry (MALDI-TOF MS) technique^15,16^. There is still no quantitative analysis of sperm fatty acids in this species.

In addition to elucidating the chemical components of sperm membranes, several studies focused on their biophysical characteristics, e.g., fluidity^17–20^ or generalized polarization ^19–22^, which describes the order of membrane lipids and membrane hydration level^23^. Both parameters are shaped by many factors, including membrane lipid content: sterol/phospholipid ratio and saturated/unsaturated fatty acids ratio^2,23^. Therefore, biophysical analysis provides an overall picture of membrane biochemical composition and allows examination of dynamic changes, e.g. during cryopreservation ^17–19,22^. To our knowledge, such an analysis has not been performed in domestic cat spermatozoa.

The aim of this study was to characterize the fatty acid content and biophysical characteristics (anisotropy-ANISO, generalized polarization-GP) of the cell membrane of domestic cat spermatozoa.

## Results

### Semen quality

The characteristics of the feline spermatozoa used in this study (Table 1) were not affected by the cat’s age or breed. Most of the cats had poor semen morphology (only two individuals had normal morphology > 60%). After cryopreservation, there was inter-individual variability in post-thaw quality, with membrane integrity (measured by flow cytometry) ranging from 18 to 68% (with 60% of males having sperm membrane integrity > 50%) and with CASA motility ranging from 8 to 61% (with 30% of males having sperm motility > 30%). Poorer results for motility were (at least partially) related to poor initial motility—more than half of the individuals retained at least 50% of pre-freeze motility.

**Table 1:** Parameters of feline semen used in different steps of this study.

	Fresh	Fresh	Frozen-thawed
	(Exp.1)	(Exp.2)	(Exp.2)
	N = 11	N = 15	N = 18
Basic semen parameters			
Subjective motility [%]	59.5 ± 29.1	73.3 ± 16.8	52.2 ± 19.7
Viability [%]	88.6 ± 9.1	87.9 ± 11.8	72.5 ± 11.1
Normal morphology [%]	32.5 ± 22.3	41.2 ± 18.2	38.9 ± 14.2
CASA parameters			
MOTILE [%]	59.9 ± 28.9	62.6 ± 17.5	36.5 ± 18.0
PROGRESSIVE [%]	33.5 ± 25.3	37.4 ± 19.5	18.2 ± 11.1
RAPID [%]	47.8 ± 25.5	52.7 ± 24.1	65.9 ± 20.6
MEDIUM [%]	23.4 ± 13.4	23.0 ± 14.1	13.9 ± 9.2
SLOW [%]	28.8 ± 17.3	24.3 ± 13.8	20.1 ± 12.4
VAP [µm/s]	119.7 ± 33.4	120.0 ± 25.7	91.7 ± 14.4
VSL [µm/s]	100.4 ± 29.5	103.0 ± 22.9	82.5 ± 14.3
VCL [µm/s]	196.9 ± 33.2	198.4 ± 27.3	155.4 ± 17.0
ALH [µm]	6.7 ± 0.7	6.8 ± 1.0	5.5 ± 0.6
BCF [Hz]	33.9 ± 3.2	35.0 ± 3.8	35.4 ± 4.3
STR [%]	82.0 ± 3.7	84.1 ± 3.9	89.7 ± 2.3
LIN [%]	50.5 ± 8.9	51.8 ± 8.2	54.0 ± 5.6
Flow cytometry assessment			
Membrane intact [%]	n/a	n/a	51.7 ± 12.8
Damaged acrosome [%]	n/a	n/a	27.8 ± 12.5
High mitochondrial potential [%]	n/a	n/a	76.9 ± 9.5

Results presented as mean ± SD. VAP, average path velocity; VSL, straight line velocity; VCL, curvilinear velocity; ALH, amplitude of lateral head displacement; BCF, beat cross-frequency; STR, straightness; LIN, linearity;

### Exp. 1 Fatty acid composition

The average sperm fatty acid (FA) content is presented in Table 2. The predominant FAs were saturated fatty acids (SFAs): palmitic acid (16:0) and stearic acid (18:0); as well as oleic acid (18:1), which itself formed almost the entire pool of monounsaturated fatty acids (MUFAs). The mean content of polyunsaturated fatty acids (PUFAs) was low (6.12 ± 5.80%), with the mean percentage of arachidonic acid (ARA, 20:4, n-6), docosapentaenoic acid (DPA; 22:5, n-6) and docosahexaenoic acid (DHA; 22:6, n-3) less than 1% each. No significant correlations were found (*p* > 0.05) between the sperm parameters and the percentage of each FA, or total SFA, MUFA, PUFA, n-3 PUFA and n-6 PUFA (Suppl. Material).

**Table 2:** Percentage composition of fatty acids found in feline spermatozoa (n = 11). Results presented as mean ± SD. SFA, saturated fatty acids (sum); MUFA, monounsaturated fatty acids (sum); PUFA, polyunsaturated fatty acids (sum).

Fatty acid	% of total
C12:0 Lauric acid	1.55 ± 1.91
C14:0 Myristic acid	3.34 ± 3.17
C16:0 Palmitic acid	45.91 ± 12.06
C16:1 Palmitoleic acid	0.16 ± 0.23
C18:0 Stearic acid	18.69 ± 6.90
C18:1 Oleic acid	20.54 ± 12.38
C18:2 Linoleic acid	3.02 ± 2.70
C18:3 Linolenic acid	0.19 ± 0.15
C20:0 Arachidic acid	0.27 ± 0.40
C20:1 Eicosenoic acid	0.21 ± 0.48
C20:2 Eicosadienoic acid	0.42 ± 0.35
C20:3 n6 Dihomo-gamma-linolenic acid	0.05 ± 0.06
C20:3 n3 Eicosatrienoic acid	0.50 ± 0.30
C20:4 Arachidonic acid	0.37 ± 0.81
C20:5 Eicosapentaenoic acid	0.04 ± 0.10
C22:1 Erucic acid	0.30 ± 0.91
C22:2 Eicosedienoic acid	1.25 ± 0.82
C22:6 Docosahexaenoic acid	0.30 ± 0.50
SFA	69.76 ± 24.45
MUFA	21.21 ± 14.00
PUFA (total)	6.12 ± 5.80
PUFA n-3	0.86 ± 0.59
PUFA n-6	5.26 ± 3.12
Ratio unsaturated:saturated	0.39 ± 0.1

### Exp. 2 Membrane fluidity and generalized polarization

**Figure 1:**
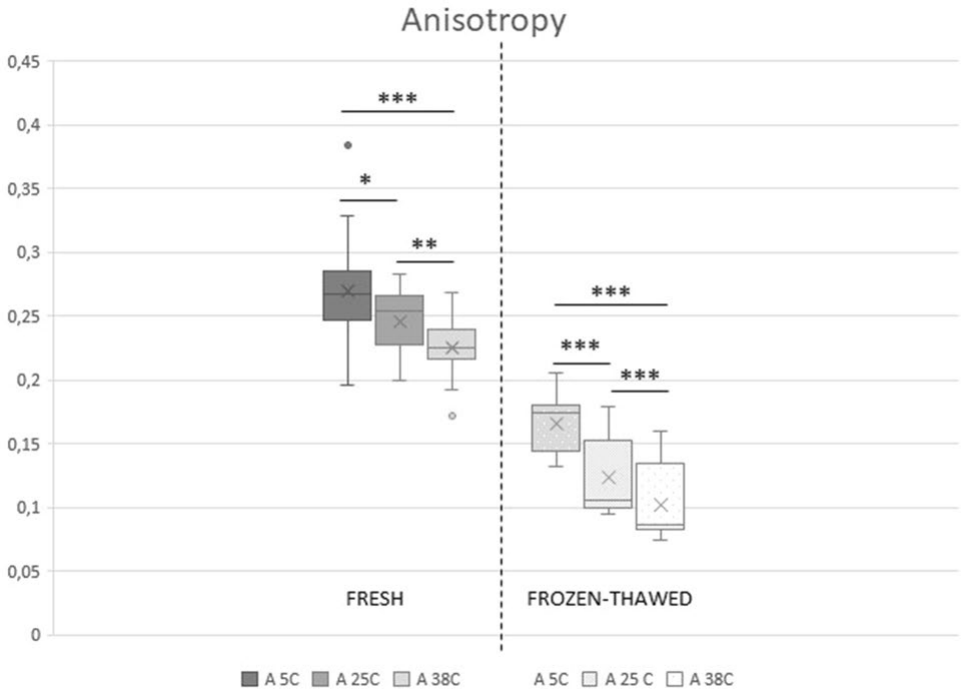
Anisotropy of sperm membranes measured at three temperature points: 38 °C (A 38C), 25 °C (A 25C), and 5 °C (A 5C), in fresh (n = 15) and frozen-thawed (n = 18) feline spermatozoa. The upper and lower sides of the box represent lower and upper quartiles, the vertical line represents the median, the cross represents the mean. The whiskers represent minimal and maximal value, circles represent outliers. Asterisks indicate significant differences: *p < 0.05, **p < 0.02, ***p < 0.001.

**Figure 2:**
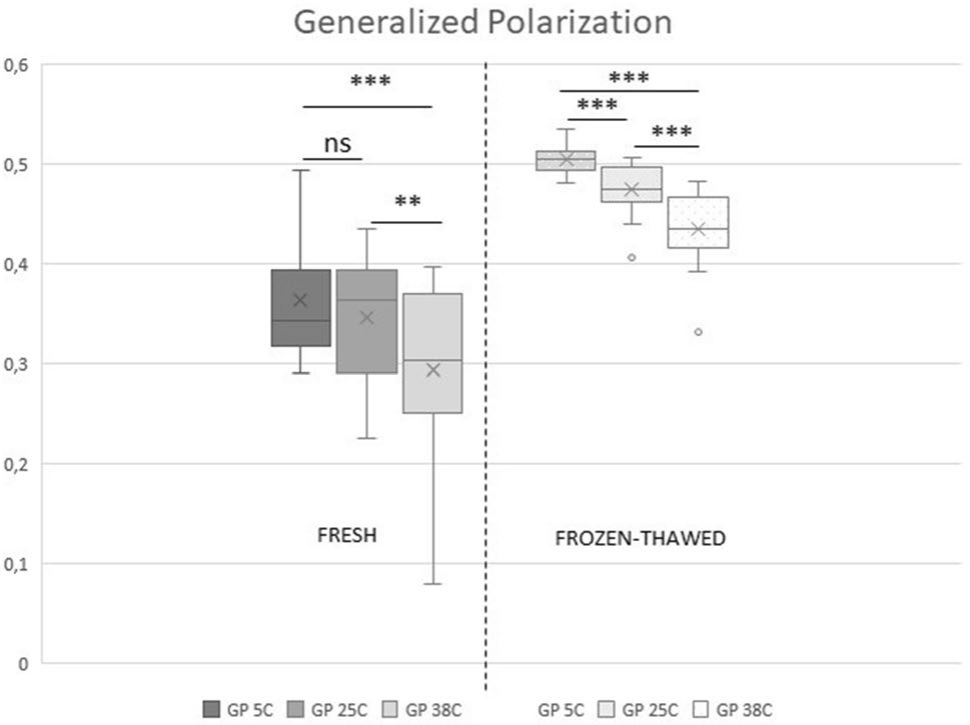
Generalized polarization of sperm membranes measured at three temperature points: 38 °C (GP 38C), 25 °C (GP 25C), and 5 °C (GP 5C), in fresh (n = 15) and frozen-thawed (n = 18) feline spermatozoa. The upper and lower sides of the box represent lower and upper quartiles, the vertical line represents the median, the cross represents the mean. The whiskers represent minimal and maximal value, circles represent outliers. Asterisks indicate significant differences: *p < 0.05, **p < 0.02, ***p < 0.001, ns—not significant.

In fresh spermatozoa, lowering the temperature from 38 to 25 °C and then to 5 °C caused a significant increase (*p* < 0.05) in mean ANISO from 0.22 ± 0.02 to 0.25 ± 0.03 and then to 0.27 ± 0.05, indicating a decrease in membrane fluidity (Fig. 1). Reducing the temperature from 38 to 25 °C significantly increased GP (from 0.29 ± 0.09 to 0.35 ± 0.06, *p* < 0.05) (Fig. 2). Further changes between 25 °C and 5 °C (to 0.36 ± 0.06) were not significant (*p* > 0.05).

Cryopreservation significantly decreased ANISO (increased sperm membrane fluidity) and GP at each temperature point (Figs. 1 and 2) (*p* < 0.05). In frozen thawed samples, changes in temperature above 0 °C were similar to those observed in fresh semen, but more pronounced (Figs. 1 and 2) (*p* < 0.05).

In fresh semen, there were generally no significant correlations between biophysical characteristics and other parameters, with a few exceptions (Suppl. Material). Cat age was strongly, positively correlated with ANISO at 38 °C (r = 0.68, *p* < 0.05) and at 25 °C (r = 0.71, *p* < 0.05). ANISO at 38 °C showed a moderate and negative correlation with BCF (r = − 0.54, *p* < 0.05), and GP at 5 °C showed a positive correlation with MEDIUM subpopulation (r = 0.54, *p* < 0.05).

In frozen-thawed semen, multiple significant correlations were found, all presented in Suppl. Material. ANISO and GP (different correlations at different temperature points, but mostly at 38 °C) were negatively correlated with several post-thaw motility parameters, but not with viability. Furthermore, there was a negative correlation between ANISO at 38 °C (r = − 0.60, *p* < 0.05) and at 25 °C (r = 0.75, *p* < 0.05) and the percentage of cells with a damaged acrosome.

When comparing biophysical membrane characteristics in fresh semen and the quality of cryopreserved semen, GP (all temperature points) and ANISO at 5 °C were not correlated with any post-thaw parameters (Suppl. Material). ANISO at 38 °C showed strong and positive correlations with subjective motility (r = 0.73, *p* < 0.05), viability assessed by eosin-nigrosin staining (r = 0.69, *p* < 0.05), total motility (r = 0.76, *p* < 0.05), progressive motility (r = 0.85, *p* < 0.05), RAPID subpopulation (r = 0.85, *p* < 0.05), VAP (r = 0.81, *p* < 0.05), and VSL (r = 0.78, *p* < 0.05). ANISO at 5 °C showed similar results: there were strong, positive correlations with progressive motility (r = 0.78, *p* < 0.05), RAPID subpopulation (r = 0.75, *p* < 0.05), VAP (r = 0.67, *p* < 0.05), and VSL (r = 0.70, *p* < 0.05).

## Discussion

The current study reports, for the first time, the quantitative fatty acid composition of domestic cat spermatozoa and the biophysical properties of the sperm membranes. This knowledge can be used to enhance our understanding of changes that occur during cryopreservation, as well as in the further optimization of freezing media and protocols.

The most interesting finding in this study is that feline spermatozoa have a very low percentage of PUFAs. This is consistent with MALDI-TOF MS analysis, where spectra for ARA, DPA, and DHA were not detected in feline electroejaculated samples^15^. A similarly low content was reported for some bat species^13^, but mammalian spermatozoa are generally described as having a high content of PUFAs^4,24^, which has multiple biological implications.

Firstly, PUFAs seem to play a crucial role in spermatogenesis, as research on knockout mouse models showed that alterations in lipid metabolism mostly led to abnormal spermatogenesis and male infertility (reviewed by^24^). There are differences in lipid content between immature germ cells and mature spermatozoa^25^, as well as remodeling of membrane lipid composition throughout epididymal maturation^6,26^. In cats, phosphatidylcholine spectra containing FA C22:5 were detected in epididymal, but not in electroejaculated, spermatozoa^15^, suggesting that PUFA content decreases during maturation in this species.

Secondly, PUFAs increase membrane fluidity, fusogenicity, and flexibility, which are essential for normal sperm function, i.e. motility, capacitation, acrosome reaction, and fusion with oocyte^3,4^. Multiple studies in humans (reviewed by^4^) showed a significantly higher content of n-3 PUFAs: alpha-linolenic acid C18:3, eicosapentaenoic acid C20:5 n-3 (EPA) and DHA in fertile men compared to infertile, whereas infertile men had a higher content of n-6 PUFAs (linoleic acid C18:2 and ARA), as well as MUFAs and main SFAs (palmitic acid C16:0 and stearic acid C18:0). In boars, PUFAs, n-3 PUFAs and DHA were positively correlated with sperm motility, viability, and normal morphology^7^. We did not find any correlations between sperm parameters and lipid content in cat spermatozoa, which may be the result of a small sample size and/or the poor sperm morphology that is typical for cats—only two cats were classified as normozoospermic (> 60% normal spermatozoa). In this context, our finding that n-6 PUFAs predominated n-3 PUFAs is very interesting, especially in terms of cat nutrition, as dietary supplementation with n-3 PUFAs changed the FA profile of spermatozoa and had a positive effect on semen quality in humans (reviewed by^27^) and multiple animal species (reviewed by^28^). Enrichment of cat food with n-3 PUFAs to improve semen morphology is worth further investigation.

On the other hand, a high PUFA content in the plasma membrane renders spermatozoa susceptible to oxidative stress^29^. Free radicals and/or reactive oxygen species target PUFAs, leading to lipid peroxidation and the generation of new radicals (lipid peroxidation chain reaction). Additionally, harmful metabolites (e.g. malondialdehyde, 4-hydroxynonenal) are produced, which destabilize the sperm plasma membrane, and decrease sperm quality and fertilizing ability^29^. Semen has an antioxidant system; however, when oxidative stress exceeds its capacity, it is detrimental to spermatozoa^30^. This situation may occur when the amount of PUFAs is too high—in boars, the highest supplementation of n-3 PUFAs increased malondialdehyde production in sperm and decreased total antioxidant capacity of seminal plasma, while reducing the positive effect on progressive motility noted with lower dose of n-3 PUFAs^31^. Similarly, in rabbits, a diet enriched in n-3 PUFAs resulted in higher reactive oxygen metabolite production, higher spontaneous acrosome reaction, and poorer semen quality after cold storage^8^. The low content of PUFAs found in this study may explain our previous findings that lipid peroxidation in domestic cat sperm is low, both in fresh semen (< 5.0%) and after cryopreservation (< 7%)^32^.

Fourthly, membrane lipid constitution is an important factor in sperm cryopreservation. After freezing–thawing, multiple changes in sperm membranes can be observed, including in lipid content. In many species, cryopreservation caused a decrease in PUFA concentration^9,33,34^, probably due to lipid peroxidation^34^. Due to the high number of spermatozoa required for fatty acid measurements, this analysis was not performed on frozen-thawed spermatozoa in this study. However, we evaluated the biophysical characteristics of the sperm membranes, which are related to lipid content and behavior. In fresh semen, membrane fluidity decreased with a reduction in temperature, which is consistent with the literature^18,19,35^. Also, the increase in GP value between 38 and 25 °C was in agreement with data for other species^22^. After cryopreservation, higher GP values were observed, similar to another study in bulls^22^, indicating a higher order of lipids in the hydrophilic domain of the bilayer, and its dehydration. Surprisingly, contrary to studies in humans^17^ and birds^18^, cryopreservation resulted in increased membrane fluidity. The rigidifying effect of sperm freezing in men and birds can be explained by changes in lipid composition (decrease in PUFA content). In cats, the percentage of PUFAs was already low, so any changes caused by cryopreservation might not have contributed to post-thaw membrane fluidity. On the other hand, a loss of cholesterol from sperm membranes after cryopreservation was also reported^33,36^. As cholesterol has a stiffening effect on the membrane at temperatures above the lipid melting point^2^, loss of cholesterol leads to increased fluidity^35^. Cholesterol content was not evaluated in this study, therefore further studies are required to confirm this hypothesis.

Another explanation could be the modification of sperm membranes by the freezing extender, as spermatozoa are able to uptake molecules from the environment, and the addition of egg yolk has been shown to modify the sperm lipid content^33,37^. Unlike the sperm cryopreservation studies in humans^17^ and birds^18^, our freezing extender contained egg yolk. Therefore, the increased fluidity observed in this study could have been due to the incorporation of some egg yolk components into the sperm membrane. Furthermore, Giraud et al.^17^ used a higher glycerol concentration than was used in this study (14% vs 6%). The authors hypothesized that the observed loss of fluidity may be due to the presence of unwashed glycerol molecules within the bilipid layer^17^, which might not be valid for our experiment. Further experiments with different freezing extenders could shed some light on this subject.

Finally, since the sperm membrane is highly affected by cryopreservation (and is at the same time indispensable for sperm survival and maintenance of fertilizing ability), its lipid content and characteristics were extensively studied in the context of variable cryotolerance of different species or individuals (freezability prediction) (reviewed by^28,38^). Comparison between closely related species showed that species in which sperm survive cryopreservation well (silver foxes, African elephants, wombats, and koalas) had higher percentages of DPA and DHA than those for which sperm freezing is challenging (blue foxes, Asiatic elephants and kangaroos)^12,14,37^. It is hard to state whether domestic cats should be classified as a species that freezes well. In our study, despite their very low PUFA content, the spermatozoa from around 60% of the individuals showed good cryopreservation results, while those from the remainder of the individuals had rather poor results. Some authors describe sperm cryopreservation in cats as more difficult than in other species^39^, although many researchers have obtained post-thaw motility > 50% (reviewed by^39^). As oxidative damage is considered to be an important cause of damage during sperm freezing^40^, the low level of lipid peroxidation in cat spermatozoa may explain their relative cryotolerance. Oxidative stress explains the hypothesis described by White^41^, according to which species with a high ratio of unsaturated:saturated FAs (2.5–3.0) are classified as susceptible to cold shock, while those with low ratio (around 1.0) are classified as less susceptible. Domestic cats with a mean ratio of 0.39 should be classified as cold-resistant. Also, factors other than PUFA content play a role, e.g., protein-to-phospholipid ratio (negative effect) and cholesterol-to-phospholipid ratio (positive effect)^5^. In feline spermatozoa, the cholesterol-to-phospholipid ratio is 0.3^16^, which is higher than the 0.26 in boars (semen hard to freeze), but lower than the 0.46 in bulls (semen easier to freeze)^5^, which may explain the relatively good cryopreservation results in cats.

In addition to inter-species comparisons, efforts have been made to explain individual variations in cryotolerance and to improve freezability. In humans, PUFAs, n-3, and especially DHA, were positively correlated with sperm motility and viability after freezing/thawing^42^. In boars, long-chain PUFAs in frozen-thawed semen were positively correlated with post-thaw viability^43^, similar to horses^11^. However, in bulls, there were only slight differences in fatty acids 22:0, 18:1 cis 9, and 14:0 between good and poor freezers^10^. Data about PUFAs are in agreement with the biophysical studies: a higher amount of unsaturated FA increases sperm membrane fluidity, and in humans, higher membrane fluidity ensures better sperm cryosurvival^17^. In this study we obtained the opposite results, which may be linked with the findings discussed above—as cryopreservation increased membrane fluidity, higher rigidity before freezing may be beneficial for feline spermatozoa. Further studies on semen preservation in cats may focus on pre-freezing modification of membrane fluidity. This can be achieved by manipulating the membrane cholesterol level, which has been studied in several animal species^19,20,35,36^.

## Conclusions

Feline spermatozoa are characterized by a low PUFA content and a low ratio of unsaturated:saturated fatty acids, which may explain the low level of lipid peroxidation in this species. Cryopreservation alters the structure of the sperm membrane, increasing the fluidity of the hydrophobic part of the lipid bilayer and the lipid order in the hydrophilic part. As lower membrane fluidity in fresh semen was linked with better viability and motility after cryopreservation, this parameter may be considered an important factor in determination of sperm cryoresistance. Further studies on other membrane components (cholesterol, proteins) are required to fully elucidate feline sperm membrane behavior during cryopreservation.

## Materials and methods

### Animals

In the study 34 semen samples were collected from 33 privately owned male cats of different breeds (27 domestic shorthairs, 2 main coons, and 2 Norwegian Forest, one British shorthair and one Persian) and age (from 7 months to 8 years, mean 2 years, the same for both experiments). Animals were patients of the Department of Reproduction and Clinic for Farm Animals of the Wroclaw University of Environmental and Life Sciences, presented for routine castration procedures or semen collection for artificial insemination or cryopreservation. Semen collection during routine veterinary procedures was approved by the Local Ethics Committee in Wrocław (decision 044/2021). All experiments were performed in accordance with the national Act on the Protection of Animals Used for Scientific or Educational Purposes (Dz. U. poz. 266) and the study is reported in accordance with ARRIVE guidelines.

### Semen collection

Semen collection was performed by urethral catheterization after administration of medetomidine hydrochloride (100 µg/kg body weight) with additional transscrotal stimulation, as previously described^44^. The semen from the catheter was immediately placed in an Eppendorf tube containing 200 µL Tris-based semen extender (3.02% (w⁄ v) Tris, 1.35% (w/v) citric acid, and 1.25% (w/v) fructose in double distilled water, pH 6.5; all reagents purchased from Sigma-Aldrich Poland) prewarmed to 37 °C.

### Experimental design

**Figure 3:**
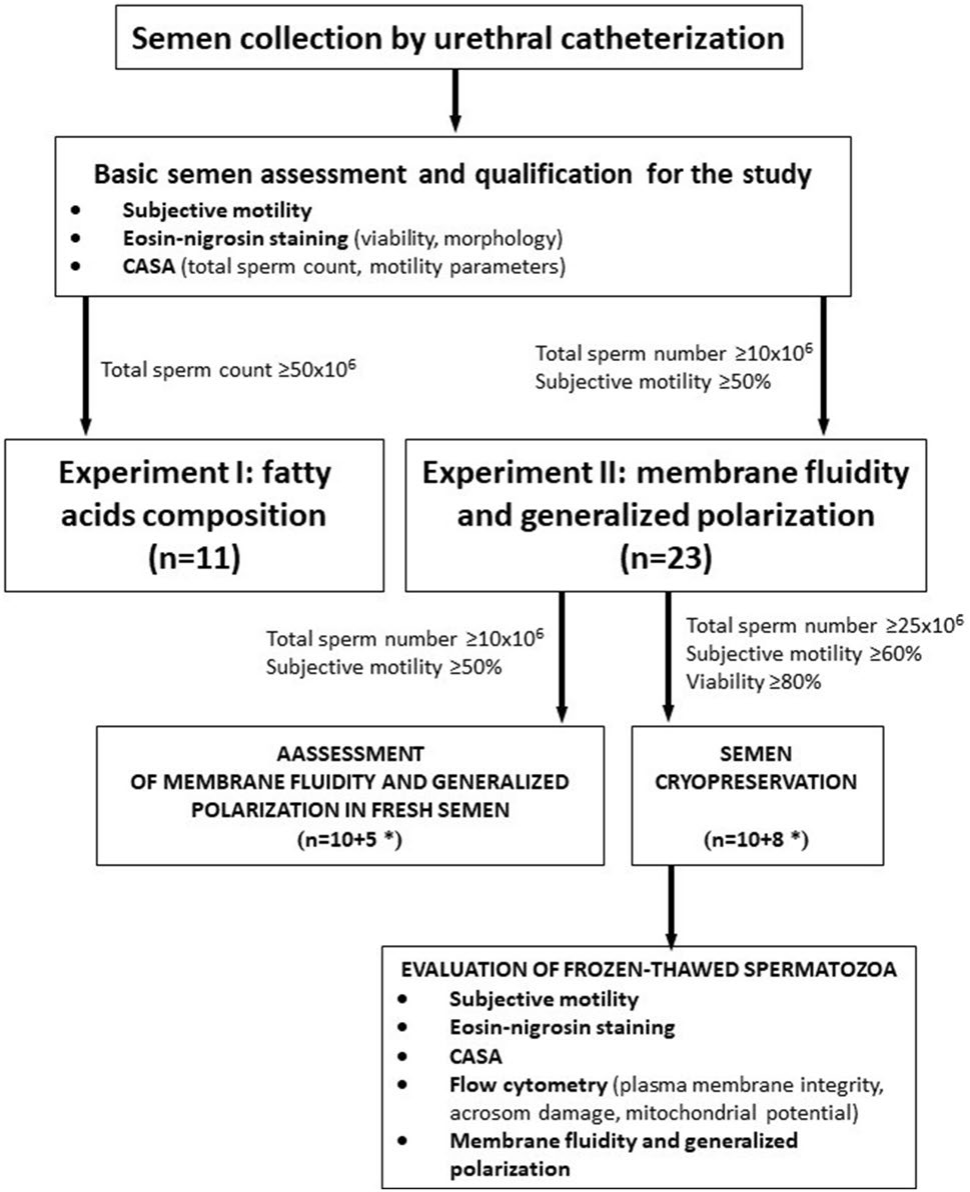
Experimental design. *ten samples were both analyzed fresh and underwent cryopreservation, five were only analyzed fresh and 8 were only cryopreserved. *CASA* computer assisted sperm analysis.

Experimental design is presented in Fig. 3. Due to the limited number of spermatozoa that it was possible to obtain from one individual (from 2.9 × 10^6^ to 93.2 × 10^6^, on average 25.9 × 10^6^^44^), the study was divided into two experiments.

### Experiment 1—Evaluation of fatty acid composition

Based on our preliminary experiments (data not shown), the minimum number of spermatozoa required for this evaluation was 50 × 10^6^. Therefore, only samples containing at least 50 × 10^6^ spermatozoa (n = 11, cats’ age from 7 months to 8 years) were included in the analysis, regardless of other quality parameters. The analysis was only performed using fresh spermatozoa. The samples were centrifuged (620×*g* for 5 min) and stored as a pellet at − 80 °C for several months until evaluation. Storage at − 70 °C was proved to be not detrimental for membrane lipids ^12^.

### Experiment 2—Biophysical analysis—membrane fluidity and generalized polarization

For biophysical analysis, another 23 semen samples (cat’s age from 7 months to 8 years) were used. Due to the insufficient quality for freezing (see below) and some technical problems, the assessment included fresh semen from 15 cats (n = 15) and frozen-thawed semen from 18 cats (n = 18), with ten of the samples analysed as both fresh and frozen-thawed. Fresh samples were evaluated within two hours after collection, and frozen-thawed samples within one hour after thawing. The frozen-thawed samples were centrifuged (620×*g* for 5 min) and the pellet was resuspended in Tris buffer to remove egg yolk particles before anisotropy and generalized polarization assessment.

### Semen assessment

#### Basic semen assessment

Immediately after collection, each semen sample was evaluated by microscopic examination (subjective motility, viability, morphology) and by computer-assisted sperm analysis (CASA). For subjective motility, 10 µl of semen was placed on the microscope slide prewarmed to 37 °C and evaluated by two independent skilled researchers using a phase contrast microscope. The mean of two assessments was calculated. Viability and morphology were assessed on eosin-nigrosin stained slides, as previously described^45^. The percentage of live spermatozoa was assessed based on 200 spermatozoa; morphology was assessed in another 200 spermatozoa. Computer-assisted sperm analysis (CASA) was carried out with an HTM-IVOS (12.3D; Hamilton-Thorne Biosciences, USA). The procedure and software settings were as previously described^45^. The following parameters were evaluated: the percentage of motile spermatozoa (MOT, %); the percentage of progressively motile spermatozoa (PMOT, %); average path velocity (VAP, µm/s); straight line velocity (VSL, µm/s); curvilinear velocity (VCL, µm/s); amplitude of lateral head displacement (ALH, µm); beat cross frequency (BCF, Hz); straightness (STR, %) and linearity (LIN, %). Based on the VAP values, subpopulations of spermatozoa showing different categories of movement were distinguished: RAPID (%) (VAP > 100 µm/s), MEDIUM (20 < VAP < 100 µm/s) and SLOW (VAP < 20 µm/s).

### Fatty acid composition

The analysis of the fatty acid (FA) content was performed as previously described^26^. FAs were extracted from whole spermatozoa, because it was reported that there are only subtle differences in their proportional distribution in the sperm plasma membrane and in whole sperm^5^. Fatty acid methyl esters (FAMEs) were prepared by incubating samples (20 μL) with 1 ml of NaOH in methanol (0.5 M) and 1 ml of BF3 (14% solution in methanol) in the presence of 0.1% (w/v) BHT. The transesterification reaction was carried out in a dry block heater (Eppendorf Poland Sp.z o.o, Warsaw, Poland) at 85 °C for 30 min. At the end of the reaction, the tubes were cooled to room temperature, briefly spun, and 1.5 mL of 0.9% (w/v) NaCl and 2 ml of n-hexane were added to the solution. The mixture was vortexed for 5 min and both organic and hydroalcoholic phases were separated by centrifugation at 1500*g* for 5 min. The upper organic (n-hexane) phase was transferred into a new glass tube. The n-hexane phases were evaporated under a nitrogen flux and the FAMEs were resuspended in 30 μL of n-hexane. FAME samples were transferred to 0.2 mL crimp vials (Agilent, Technologies, Santa Clara, United States) prior to injection into the gas separator to perform gas chromatography.

The FAMEs were analysed using a gas chromatograph coupled to a flame ionisation detector (Agilent 7890A). FAME samples dissolved in n-hexane were introduced into the injection port heated to 250 °C with an automated liquid sampler (Agilent 7650A). Samples were injected without splitting. The FAMEs were separated on a 30-m long × 0.25-mm ID × 0.25 μm DB-23 capillary column (Agilent) using He as vector gas (2.6 mL/min). The oven temperature, initially set to 100 °C for 2 min, was first increased to 160 °C at 25 °C/min, then to 250 °C at 8 °C/min and maintained at this temperature for an additional 4 min. The detector temperature was set to 270 °C while the detector gases were set to 30 mL/min for H2, 400 mL/min for air, and 30 mL/min for makeup gas (He). Data were recorded at a frequency of 50 Hz. FAME quantifications were performed with calibration curves built with the Supelco 37 component FAMEs mix (Sigma-Aldrich) using 17:0 methyl ester as the internal standard (250 ng). The determination of FAMEs was based on the retention times of each component and compared with those of FAMEs contained in the Supelco 37 FAME mix (Sigma-Aldrich) and of 22:5 n-3 and 22:5 n-6.

### Biophysical analysis: membrane fluidity and generalized polarization

Both parameters were evaluated as previously described for chicken spermatozoa^19^. Membrane fluidity was assessed by measuring fluorescence anisotropy (ANISO) with the fluorescent dye 1,6-diphenyl-1,3,5-hexatriene (DPH) inserted into the hydrophobic lipid fraction of the plasma membranes. The samples were suspended in 2 ml of Tris–EDTA diluent at a sperm concentration of 2.5 × 10^6^/mL with a DPH working solution (2.5 µM prepared from a DPH stock solution of 2 mM in dimethyl sulfoxide (DMSO)) in quartz cuvettes. DPH uptake did not increase the percentage of nonviable cells and did not affect sperm motility. Measurements were performed with a fluorimeter (CARRY Eclipse, VARIAN, California, USA) at 38 °C, 25 °C, and 5 °C. Excitation and emission wavelengths were λ_ex_ = 360 nm and λ_em_ = 425 nm, respectively. The fluorescence anisotropy (ANISO) of the DPH probe was calculated using the formula:

$$ANISO=\frac{({I}_{II} - {GI}_{\perp })}{( {I}_{II}+ {2GI}_{\perp })}$$

where I_II_ and I_┴_ = fluorescence intensities observed in directions parallel and perpendicular, respectively, to the polarization direction of the exciting wave. G is an apparatus constant dependent on the emission wavelength. Fluorescence anisotropy is inversely proportional to membrane fluidity; therefore, the lower the ANISO value, the higher the fluidity.

Generalized polarization (GP) of the membrane reflecting the arrangement of the hydrophilic part of the membrane, was assessed on the basis of changes in the fluorescence intensity of the Laurdan probe (6-dodecanoyl-2-dimethylaminonaphthalene), which was calculated from the formula:

$$GP=\frac{({I}_{b} - {I}_{r})}{({I}_{b} + {I}_{r})}$$

where I_b_ = fluorescence intensity at λ = 440 nm, I_r_ = fluorescence intensity at λ = 490 nm. Samples were suspended in 2 ml of Tris–EDTA diluent at a sperm concentration of 2.5 × 10^6^/ml with Laurdan working solution (2.5 µM prepared from a Laurdan stock solution of 2 mM in dimethyl sulfoxide (DMSO)) in quartz cuvettes. The measurements were conducted with the fluorimeter (CARRY Eclipse, VARIAN) at 38 °C, 25 °C, and 5 °C. The excitation wavelength was 360 nm and the emitted fluorescence was recorded at two wavelengths, 440 and 490 nm.

The higher the value of GP, the more ordered the hydrophilic part of the membrane.

### Flow cytometry assessment

The number of spermatozoa in most of the samples was insufficient to perform flow cytometry assessment in both fresh and frozen-thawed spermatozoa. As we were more interested in changes caused by semen freezing, only cryopreserved samples were analyzed with this technique. Analysis was performed as previously described^32^. Sperm samples were diluted to a concentration of 10 × 10^6^/mL. Measurements were carried out on a FACSCalibur flow cytometer (Becton Dickinson, San Jose, CA, USA). The fluorescent probes were excited by an argon ion 488 nm laser. Detection of green fluorescence (dyes: SYBR-14, PNA-Alexa Fluor^®^ 488 conjugate, monomer form of JC-1) was set with an FL1 bandpass filter at 530/30 nm. Orange fluorescence (J-aggregate form of JC-1) was identified in the FL2 filter band-pass filter at 585/42 nm. Red fluorescence (propidium iodide (PI)) was identified on the FL3 longpass detector at > 670 nm. Acquisitions were measured using CellQuest 3.3 software (Becton Dickinson, San Jose, CA, USA). Non-sperm events were gated out based on scatter properties and not analyzed. A total of 5,000 events were analyzed for each sample; each sample was run in one replicate.

Plasma membrane integrity was assessed using dual fluorescence staining with SYBR-14 and PI (Live/Dead Sperm Viability Kit, Life Technologies Ltd, Carlsbad, California, USA). Two and a half µL of SYBR-14 working solution was added to 300 µL of semen samples. The working solution was obtained by diluting a commercial solution of SYBR-14 in TRIS buffer with a ratio of 1:49. The samples were incubated at room temperature in the dark for 10 min. Cells were counterstained with 1.5 µL of PI immediately before analysis. Sperm cells that showed green fluorescence (SYBR-14 positive) were considered membrane intact (live), those that showed red fluorescence (PI positive) as membrane damaged (dead), and those showing green and red fluorescence as moribund.

Acrosome damage was assessed using lectin PNA (PNA of Arachis hypogaea Alexa Fluor® 488 conjugate, Life Technologies Ltd, Carlsbad, California, USA) and PI. Briefly, 5 µL of FITC-PNA working solution (1 µg/mL) was added to 500 µL of semen samples and incubated for 5 min at room temperature in the dark. The samples were centrifuged (620×*g* for 5 min), the supernatant was removed, and the sperm pellets were resuspended in 500 µL of Tris buffer. For counterstaining, 1.5 µL of PI was added to the samples immediately before cytometric analysis. Sperm cells showing green fluorescence (PNA positive) were considered acrosome damaged, regardless of their viability (PI stained—dead, non-stained—live).

Sperm mitochondrial activity was determined by staining with JC-1 (Life Technologies, Ltd, Carlsbad, California, USA) and PI. Briefly, 0.35 µL of JC-1 stock solution (3 mM JC-1 in DMSO) was added to 500 µL of the sperm sample. The samples were incubated at 37 °C in the dark for 20 min. Then 1.5 µL of PI was added to the samples prior to cytometric analysis. Dead spermatozoa (PI stained) were gated out. Spermatozoa that emitted predominantly orange fluorescence (J-aggregate form of JC-1) were classified as having high mitochondrial activity, and those that emitted mostly green fluorescence (monomer form of JC-1) as having low mitochondrial activity.

### Semen cryopreservation

Samples showing satisfactory basic quality (total sperm count at least 25 × 10^6^ spermatozoa, motility at least 60%, viability at least 80%) were subjected to cryopreservation, according to the protocol for feline semen used routinely in our laboratory^32^. Briefly, samples were centrifuged at 620×*g* for 5 min, then the supernatant was removed, and the sperm pellet resuspended at room temperature in a freezing extender containing Tris buffer supplemented with 20% (v/v) egg yolk, 6% glycerol (v/v) and 1% Equex paste (v/v) (Minitube, Germany). Sperm samples were loaded into 0.25 mL straws (10 × 10^6^ spermatozoa each), cooled to 5 °C within 1 h and then frozen in liquid nitrogen vapour for 10 min. The straws were plunged into liquid nitrogen and kept at − 196 °C until further evaluation. After at least one week of storage, two straws from each sample were thawed by immersion in a 37 °C water bath for 30 s and the contents were emptied into an Eppendorf tube. Basic semen analysis (subjective motility, viability, morphology) and CASA were performed in the thawed samples in the same way as for the fresh samples.

### Statistical analysis

The data distribution was assessed using the Shapiro–Wilk test. For most parameters (except age, subjective motility, viability, VAP and VSL in fresh semen, and ANISO at 25 °C, ANISO at 38 °C, subjective motility, RAPID and SLOW subpopulations in cryopreserved semen and lipid content), the data were distributed normally, therefore, all the results are presented as mean ± SD.

The ANOVA (or Kruskal–Wallis ANOVA for data not normally distributed) was used for comparison of ANISO and GP values at different temperatures. The pairwise student t-test (or Wilcoxon signed-rank test for data not normally distributed) was used to compare ANISO and GP values at given temperature points in fresh vs. frozen-thawed semen. The Pearson correlation (or the Spearmen correlation for data not normally distributed) between all parameters in fresh and cryopreserved spermatozoa was assessed and the results are provided as Supplementary Material. The statistical software Statistica v13.3 for Windows (StatSoft Polska Sp. z o.o., Cracow, Poland) was used for the analysis and the significance level was set at *p* < 0.05.

### Supplementary Information

Supplementary Tables.

## Data Availability

The data that support the findings of this study are available from the corresponding author [Sylwia Prochowska], upon reasonable request.
