# Author Correction: Fatty acid composition and biophysical characteristics of the cell membrane of feline spermatozoa

**DOI:** 10.1038/s41598-024-62067-2

**Published:** 2024-05-15

**Authors:** Sylwia Prochowska, Dorota Bonarska-Kujawa, Łukasz Bobak, Maria Eberhardt, Wojciech Niżański

**Affiliations:** 1 Department of Reproduction and Clinic of Farm Animals, Wrocław University of Environmental and Life Sciences, 50-366 Wrocław, Poland; 2 Department of Physics and Biophysics, Wrocław University of Environmental and Life Sciences, 50-375 Wrocław, Poland; 3 Department of Functional Food Product Development, Wrocław University of Environmental and Life Sciences, 51-630 Wrocław, Poland

Correction to: *Scientific Reports* 10.1038/s41598-024-61006-5, published online 03 May 2024

The Acknowledgments section in the original version of this Article was incomplete.

“The study was financed by a Polish National Center of Science Grant: NCN 2019/35/D/NZ3/02533. The authors are grateful to Mrs. Barbara Smalec for her excellent technical assistance, to Mr. Bartosz Czech for his help in statistical analysis and to dr Sharon Mortimer for the linguistic correction of the manuscript.”

now reads:

“The study was financed by a Polish National Center of Science Grant: NCN 2019/35/D/NZ3/02533. The authors are grateful to Mrs. Barbara Smalec for her excellent technical assistance, to Mr. Bartosz Czech for his help in statistical analysis and to dr Sharon Mortimer for the linguistic correction of the manuscript. The APC is co-financed by Wrocław University of Environmental and Life Sciences.”

The original Article has been corrected.

